# Eosinophilic colitis in a boy with a novel *XIAP* mutation: a case report

**DOI:** 10.1186/s12887-020-02075-z

**Published:** 2020-04-18

**Authors:** Jiamei Tang, Xiaoying Zhou, Lan Wang, Guorui Hu, Bixia Zheng, Chunli Wang, Yan Lu, Yu Jin, Hongmei Guo, Zhifeng Liu

**Affiliations:** 1 Department of Gastroenterology, Children’s Hospital of Nanjing Medical University, No. 72 Guangzhou Road, Nanjing, Jiangsu Province, 210008 China; 2 Nanjing Key Laboratory of pediatrics, Children’s Hospital of Nanjing Medical University, No. 72 Guangzhou Road, Nanjing, Jiangsu Province, 210008 China

**Keywords:** XIAP deficiency, Eosinophilic colitis, Gene detection

## Abstract

**Background:**

X-linked inhibitor of apoptosis (XIAP) deficiency is a rare primary immunodeficiency disease characterized by haemophagocytic lymphohistiocytosis, recurrent splenomegaly and inflammatory bowel disease (IBD). The only curative treatment is haematopoietic stem cell transplant (HSCT).

**Case presentation:**

Here, we report the case of a 22-month-old male with a long history of abdominal distension and anaemia. Clinical and laboratory findings were consistent with eosinophilic colitis. To identify the underlying disease, we performed exome sequencing, which showed an unreported frameshift mutation in the *XIAP* gene.

**Conclusion:**

We present eosinophilic colitis as the initial manifestation of XIAP deficiency for the first time in this article, which expands the mutation spectrum and phenotype of this disease.

## Background

X-linked inhibitor of apoptosis (XIAP) deficiency, also known as X-linked lymphoproliferative syndrome type 2 (XLP-2), is a rare inherited disease caused by a gene mutation in *XIAP*, which is an important inhibitor of programmed cell death or apoptosis by blocking the activation of caspases 3, 7 and 9. The most commom characterization of XIAP deficiency is a key triad of clinical manifestations, including a high susceptibility to developing haemophagocytic lymphohistiocytosis (HLH) frequently triggered by Epstein–Barr virus (EBV) infection, recurrent splenomegaly and inflammatory bowel disease (IBD) with the features of Crohn’s disease. To the best of our knowledge, the association of eosinophilic colitis with XIAP deficiency has not been reported in any literature to date. Here, we present the first case of XIAP deficiency complicated by eosinophilic colitis, expanding the clinical phenotype of XIAP deficiency.

## Case presentation

A 22-month-old male was admitted to our Department of Gastroenterology because of a more than one-year history of abdominal distension with anaemia. His symptoms of abdominal distension was obvious after eating and improved slightly after defecation and flatulence. Meanwhile, the patient’s stool appeared yellow and loose with visible food residue and was voided twice to four times a day.

In the past, the patient was once admitted to the local hospital with cervical lymphadenectasis and mild anaemia. At that time, he received oral iron treatment at home, but his abdominal distension showed no remission. The patient’s first admission to our hospital was due to recurrent fever for over half a year in May 2018, and he was diagnosed as bronchopneumonia with pleural effusion and liver and cardiac damage according to the examination results. The patient was born through a full-term natural delivery and showed no abnormalities in the perinatal period. He had repetitive respiratory tract infections, and fever always occurred after vaccination. Perianal abscess resection was performed at the ages of 6 months and 8 months. His parents and sister were healthy.

Physical examination indicated that the patient’s weight was 10 kg (z score: − 1.55), and his height was 80 cm (z score: − 2.05). Hardened, enlarged lymph nodes were found on the neck and bilateral sides of the groins. Abdominal distention extended from the xiphoid to the bilateral sides of the groins where the vena epigastrica was exposed. The patient’s abdominal circumference was 50.5 cm. The soft liver and spleen extended 4 cm below the rib cage. We also found that he had two surgical scars because of a perianal abscess.

**Fig. 1:**
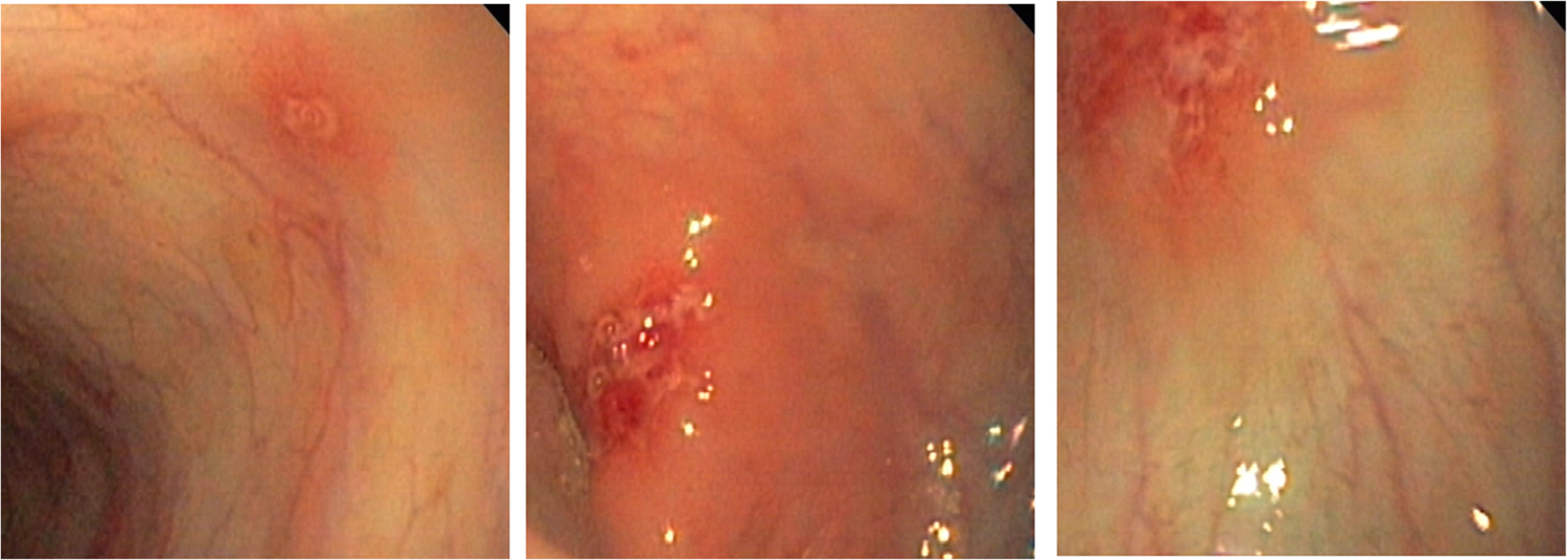
Endonoscopy revealed scattered ulcers and erosions in the intestines of the patient with XIAP deficiency

**Fig. 2:**
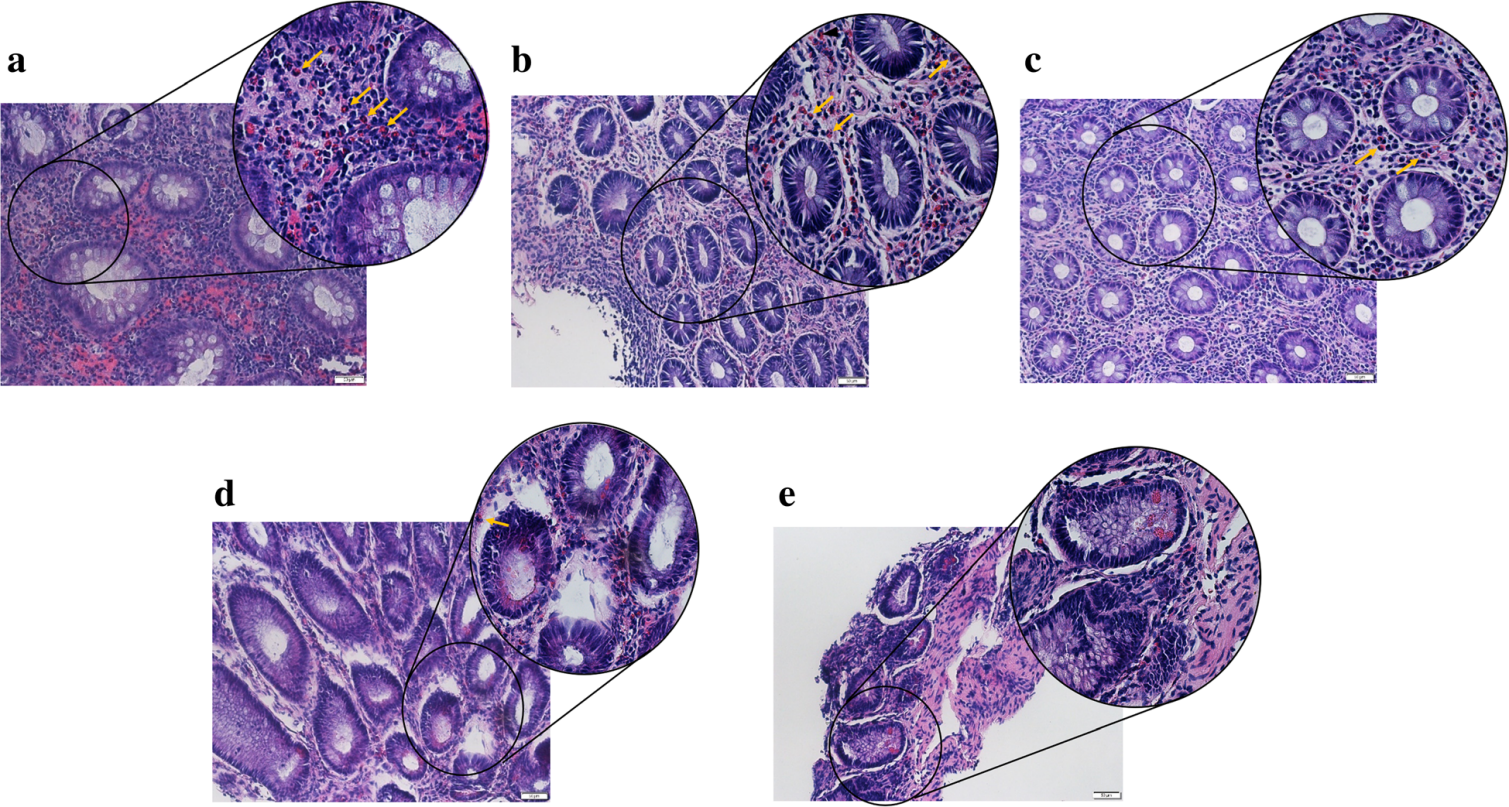
The pathology of colonoscopy showed that there was chronic active enteritis with different degrees of eosinophils infiltration in transverse colon, sigmoid colon and rectum, and there was chronic enteritis in descending colon and ileocecal valve. Numerous eosinophils (about 80/HPF) populate the lamina propria (arrow) in the section of transverse colonic (Fig. 2a, HE× 200, HE× 400 in the circle) and rectal (Fig. 2b, HE× 200, HE× 400 in the circle) mucosa. And small amount of eosinophils (about 25/HPF) infiltrate in lamina propria (arrow) in the section of sigmoid (Fig. 2c, HE× 200, HE× 400 in the circle) mucosa. A few eosinophils (about 10/HPF) populate the lamina propria (arrow) in the section of descending colon mucosa (Fig. 2d, HE× 200, HE× 400 in the circle). Only several eosinophil infiltrate in the mucosa of ileocecal valve (Fig. 2e, HE× 200, HE× 400 in the circle)

**Fig. 3:**
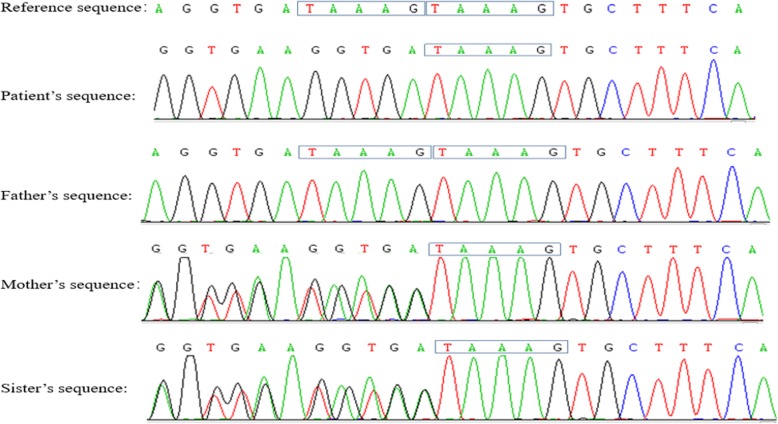
Genetic test results of their familiy showed that the *XIAP* gene sequence of the father was normal, but the patient, his sister and mother had frame-shift mutation:c.888(exon3)-c.892(exon3) del TAAAG chrX:123022479–123,022,483. p.Asp296Aspfs*12(NM:001167.3, Reference genome: Hg19)

**Fig. 4:**
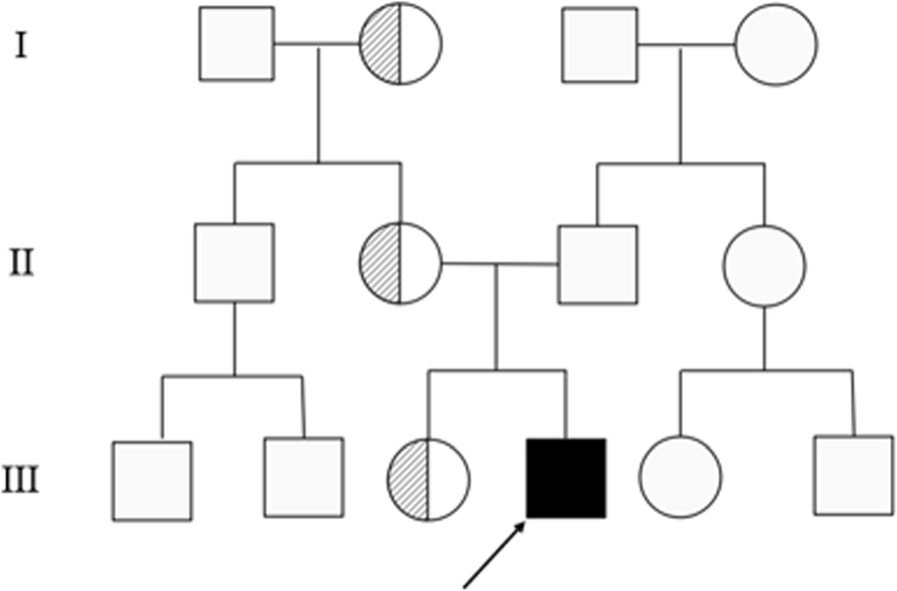
The patient’s family tree

The abdominal CT suggested hepatosplenomegaly, a slightly thickened and strengthened intestinal wall of part of the abdomen (the left abdomen was more obvious), and multiple lymph nodes in the mesenteric, retroperitoneal and bilateral inguinal region (Fig. 6, in the Supplementary file). The patient’s routine examination results are shown in Table 1. His bone marrow aspiration revealed that the proliferation of granulocytes, erythroid cells and megakaryocytes was obviously active, and platelet clusters could be seen, ruling out haematological diseases. To determine the cause of abdominal distension, we performed endoscopy and two colonic biopsies were taken from different parts of the intestine, which identified mucous hyperaemia in the membrane with ulcers and erosions from the ileocaecum to the rectum (Fig. 1). The pathologic changes are shown in Fig. 2. However, the patient began to present fever with a temperature peak of 39.5 °C on the third day after endoscopy; treatment with meropenem controlled this condition of fever, but his gastrointestinal symptoms did not improve. Considering that he may have immunodeficiency, we tried the empiric treatment of immunoglobulin replacement. To identify the underlying disease, exome sequencing was performed after obtaining written informed consent from the patient’s parents. A novel frameshift mutation c.888-892delTAAAG (p. Asp296Aspfs*12) in exon 3 of the *XIAP* gene was identified (Fig. 3). This deletion results in a shift in the reading frame and formation of a premature stop codon at the 888–892 position of the DNA strand, which corresponds to the 296-protein chain codon. As a result, peptide breakdown occurs earlier than the normal, which indicates the pathogenicity of this mutation. The patient’s healthy mother and sister were heterozygous carriers (Fig. 4). For future monitoring, we recommend that the patient should conduct regular hospital follow-up, recheck the gastroenteroscopy regularly to observe the progression of gastrointestinal inflammation and injury, and histopathological examination will be conducted to keep abreast of the progress of the disease. However, his parents decided to pursue no further therapy (including HSCT) because of the expense, and the patient is currently experiencing recurrent infections again and undergoing follow-up at the outpatient clinic.

**Table 1:** The patient’s routine inspections results during hospitalization

Haematological values (normal value)	Feces Examination (normal value)	Immunological test (normal value)
CRP:26 mg/L(0-10 mg/L) Hb:73 g/L(110-160 g/L) Eosinophil count:0.78 × 10^9/L(0.05–0.5 × 10^9/L)	Pyocyte: +++/HP RBC:1–3/HP	IgG10.6 g/L (5.09–10.09 g/L) IgM0.677 g/L(0.98–1.78 g/L) IgA0.861 g/L (0.31–0.67 g/L)
PCT:1.69 ng/ml(<0.05 ng/mL) Serum ferritin 375.3 ng/ml(11–306.8 ng/mL)	Fecal calprotectin:1295.5μg/g (<80μg/g)	NK cells 4.05% (6–27%)
Serum biochemistrytargets: AST 63 U/L(5-40 U/L); LDH 449 U/L(80-285 U/L); TRIG 1.91 mmol/L(0.56–1.69 mmol/L); HDL 0.65 mmol/L(0.78–2.0 mmol/L)	Clostridium difficile glutamic dehydrogenase antigen: positive Clostridium difficile toxin: negative	Anti-nuclear antibody IgG: weakly positive; IBD antibody: anti-intestinal goblet cell antibody weakly positive; Anti-neutrophil cytoplasmic antibody perinuclear type: weakly positive
Four items of anemia screening: erythrocyte folic acid 2120.2 nmol/L (317–1894 nmol/L); plasma erythropoietin 27.89 IU/L (2.59–18.5 IU/L)	Fecal bacterial culture:negative	
EBV-IgG:positive; EB-DNA:< 5.0E+ 2 copies/ml(< 5.0E+ 2 copies/ml) CMV-DNA:< 5.0E+ 2 copies/ml(< 5.0E+ 2 copies/ml) Parasite set:negative Blood bacterial culture:negative		

*CRP* C reactive protein; *Hb* Hemoglobin; *PCT* Procalcitonin; *AST* Aspartate transaminase;

*LDH* Lactatedehydrogenase; *TRIG* Triglyceride; *HDL* Highdensitylipoprotein; *EBV* Epstein–Barr virus; *CMV* Cytomegalovirus; *RBC* Red blood cell; *IgG* immunoglobin G; *IgM* Immunoglobin M; *IgA* Immunoglobin A; *NK cells* Natural killer cells; *IBD* Inflammatory bowel disease

## Discussion and conclusions

XIAP deficiency (OMIM 300635), also called X-linked lymphoproliferative syndrome type 2 (XLP-2), is a rare inherited disease caused by mutations in the *XIAP* gene, which encodes an important inhibitor of programmed cell death or apoptosis by blocking the activation of caspases 3, 7 and 9 and is related to signal transduction and activation processes, such as the NF-ĸB, MAPK pathway, NLRS, copper metabolism and autophagy [1]. Different patients may develop variable clinical phenotypes, including HLH, colitis or IBD, granulomatous lymphocytic interstitial lung disease (GLILD), granulomatous hepatitis, uveitis and juvenile idiopathic arthritis [2–4].

Based on our patient’s examination results, we searched relevant case reports with the keyword “XIAP” in PubMed and summarized the clinical phenotypes of XIAP deficiency. Thirty-two cases with complete clinical data were reported from 2010 to 2019 (Table 2). Most XIAP deficiency patients presented with HLH (45.45%), followed by IBD (33.33%), and fewer with uveitis (6.06%), juvenile idiopathic arthritis (3.03%), GLILD and granulomatous hepatitis (3.03%), hypogammaglobulinemia (3.03%), Langerhans cell histiocytosis (LCH) (3.03%) and asymptomatic (3.03%). However, different studies may be different in this respect. For example, a review found that 16% of XIAP patients showed hypogammaglobulinemia in five countries, including France, England, Germany, USA and Japan as of 2014 [1]. Our literature review showed that about 3% of patients showed hypogammaglobulinemia from 2010 to 2019. This is a significant discrepancy, which results from the difference of the included case samples and ages covered by the document retrieval. We found that the complication of eosinophilic colitis in patients with XIAP deficiency, which is characterized by abdominal distension, was not reported so far. Some patients can exhibit different complications, such as LCH complicated with HLH and Crohn’s disease complicated with anterior uveitis [5, 26]. Patients with XIAP deficiency may have a variety of coincident symptoms of complications, and most (including our patient) exhibit recurrent fevers and refractory infections. Positive signs of hepatosplenomegaly occur in all patients, including our patient, and anaemia, liver function and coagulation abnormalities ensue. Our patient also showed abdominal distension from the age of 1 month, which to our understanding was caused by poor intestinal digestive function related to colitis. This finding suggests that children who have abdominal symptoms as the first manifestation and are prone to repeated infections with partial clinical manifestations of HLH (but not in accordance with the criteria for HLH-2004) should cause clinicians to be alert to XIAP deficiency complicated with eosinophilic colitis, and early gene detection should be carried out. More than one hundred mutations in the *XIAP* gene have been reported in the Human Gene Mutation Database (HGMD). The frameshift mutation p. Asp296Aspfs*12 of our patient was not previously described in any literature or database. This is the first reported case of eosinophilic colitis occurring with a background of XIAP deficiency. Our empirical treatments, such as high-grade antibiotics and immunoglobulin replacement, temporarily relieve symptoms in children but did not cure the present patient. Unfortunately, the optimal treatment for this child is unknown, and there remains no established standard of care for management. The only curative treatment for XIAP deficiency is HSCT.

**Table 2:** Literature retrieval: clinical phenotypes frequency of XIAP deficiency

Disease phenotype	Case number (%)	Mean age of onset (months)	Clinical feature
IBD [5–14]	*n* = 11(33.33)	46.27	Prolonged fever、abdominal pain、diarrhea with or without blood and mucus、growth failure、perianal desease、anemia、recurrent infection、hepatosplenomegaly
HLH [15–24]	*n* = 15(45.45)	28.79	Persistent fever、pancytopenia、EBV infection、hepatosplenomegaly、lymphadenopathy
GLILD and granulomatous hepatitis [2]	*n* = 1(3.03)	240	Progressive cough、dyspnea
Uveitis [3, 5]	*n* = 2(6.06)	144	Decreased visual acuity、arthralgias、abdominal pain、weight loss、sinus infection
Juvenile idiopathic arthritis [4]	*n* = 1(3.03)	60	Prolonged fever、pancytopenia、generalized macular rash、periodic abdominal pains
Hypogammaglobulinemia [25]	*n* = 1(3.03)	N	Hypoimmunity
Langerhans cell histiocytosis [26]	*n* = 1(3.03)	13	Recurrent ear discharge、fever、 hemorrhagic papules、 hepatosplenomegaly
Asymptomatic [25]	*n* = 1(3.03)	N	No clinical manifestation

*IBD* Inflammatory Bowel Disease, *HLH* Hemophagocytic lymphohistiocytosis, *GLILD* granulomatous lymphocytic interstitial lung disease, *EBV* Epstein–Barr virus, N: not mentioned

Eosinophilic colitis is a type of eosinophilic gastrointestinal characterized by pathologic eosinophilic infiltration of the colon leading to organ dysfunction and clinical symptoms including (but not limited to) nausea, vomiting, diarrhoea, abdominal pain, abdominal distention, weight loss, and isolated ascites. IBD is part of the differential diagnosis of eosinophilic colitis in clinical work due to overlapping symptoms. Further association between XIAP deficiency and IBD has been established through epidemiologic studies, as about 4% of male patients with early onset IBD have been found to have a mutation in *XIAP* and the majority of *XIAP* variants are associated with a selective defect in NOD1/2 signalling, impaired NOD1/2-mediated activation of NF-κB, and altered NF-κB-dependent cytokine production [27]. Therefore, we hypothesized that an abnormality of the NOD2 signalling pathway may be related to the occurrence of XIAP deficiency with eosinophilic colitis. Genome-wide transcript profiles have revealed that levels of TH2 cytokines (e.g., IL4, IL5, and IL13) and the eosinophil-related chemokine eotaxin-3 (CCL26) are upregulated in eosinophilic gastrointestinal tissue [28]. Therefore, we speculate that the Th2-type immune response, which is a downstream pathway of NOD2, can be involved in eosinophilic colitis. Based on these speculative theories, some experimental data need to be supplemented, and more clinical cases and experiments are needed.

In summary, we report for the first time a boy of Chinese origin with a novel *XIAP* mutation who presented the feature of eosinophilic colitis. Our findings extend the phenotypic spectrum of XIAP deficiency. From this case review, we speculated that early detection of clinical symptoms is challenging in patients with XIAP deficiency, contributing to diversity of and difficulty in distinguishing among clinical symptoms. Therefore, we should be alert to XIAP deficiency with eosinophilia in view of repeated immunosuppression combined with long-term and intractable abdominal symptoms.

## Supplementary information

**Additional file 1.** Fig. 5 Esophagogastroduodenoscopy showed no abnormality in esophagus, stomach and duodenum. (A: esophagus; B: preventriculus; C: fundus of stomach; D: Gastric body; E: gastric angle; F: gastric antrum; G: duodenal bulb; H: descending part of duodenum).

**Additional file 2.** Fig. 6 The abnormal CT findings of the patient. A: The axial image of contrast-enhanced computed tomography scan shows hepatosplenomegaly (asterisk) and intestinal wall thickening (arrow);B: Axial unenhanced computed tomography shows slightly enlarged mesenteric lymph nodes (arrow); C:The axial image of computed tomography scan shows intestinal wall thickening suspicious (arrow); D:Computed tomography with intravenous contrast comfirms the thickened intestinal wall (arrow).

## Data Availability

Data sharing is not applicable to this article, as no datasets were generated or analysed during the current study.

## Abbreviations

XIAP: X-linked inhibitor of apoptosis
XLP-2: X-linked lymphoproliferative syndrome type 2
HSCT: Haematopoietic stem cell transplant
HLH: Haemophagocytic lymphohistiocytosis
EBV: Epstein–barr virus
IBD: Inflammatory bowel disease
GLILD: Granulomatous lymphocytic interstitial lung disease
LCH: Langerhans cell histiocytosis
HGMD: The human gene mutation database
