# Supplementary figures and images for: Eosinophilic colitis in a boy with a novel XIAP mutation: a case report
Source: BMC Pediatr. 2020 Apr 18;20:171. doi: 10.1186/s12887-020-02075-z (PMC7165398; doi:10.1186/s12887-020-02075-z)

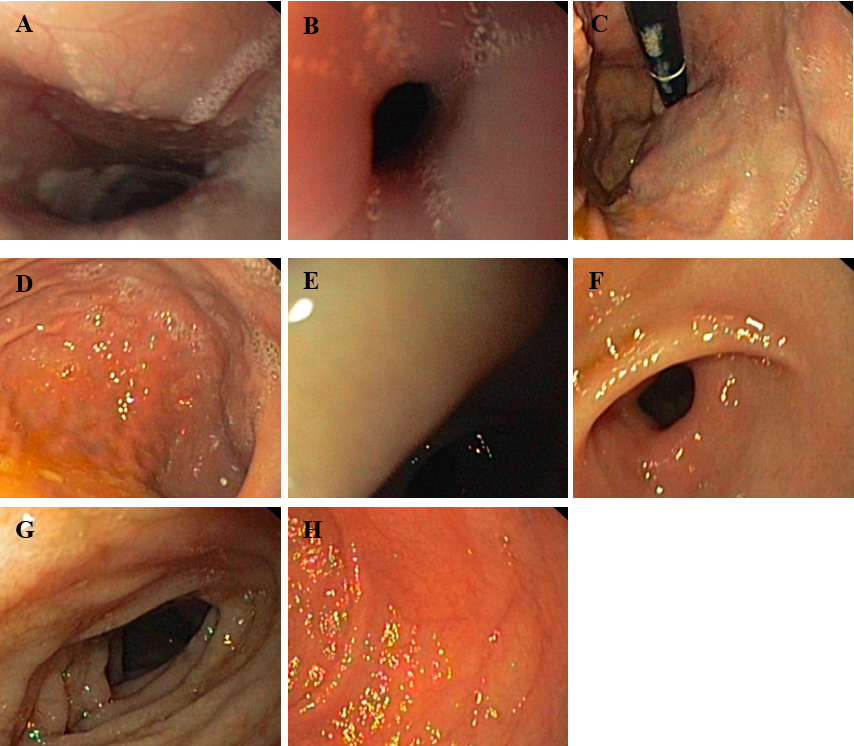

Supplement: Supplementary file 1 — Additional file 1. Fig. 5 Esophagogastroduodenoscopy showed no abnormality in esophagus, stomach and duodenum. (A: esophagus; B: preventriculus; C: fundus of stomach; D: Gastric body; E: gastric angle; F: gastric antrum; G: duodenal bulb; H: descending part of duodenum). [file 12887_2020_2075_MOESM1_ESM.tif]

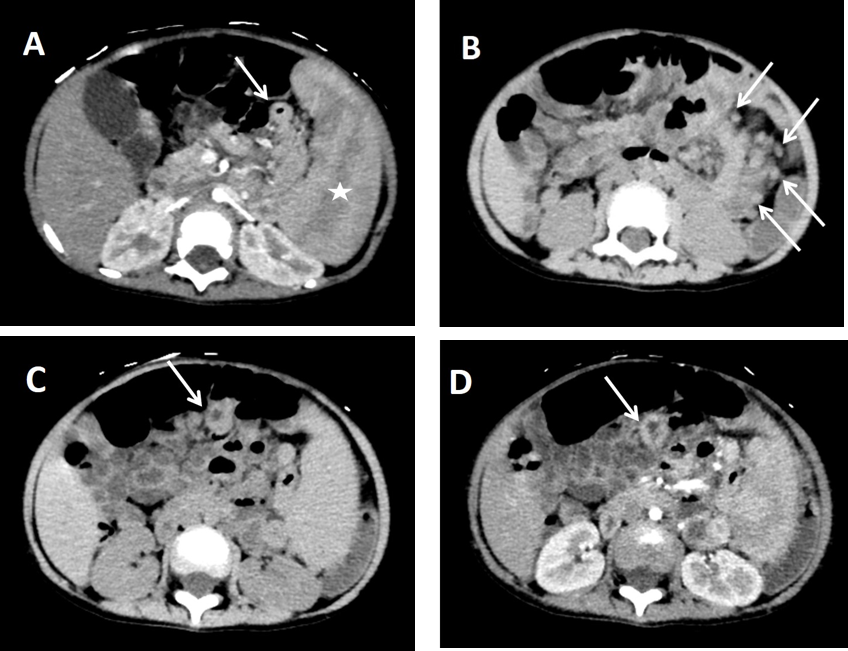

Supplement: Supplementary file 2 — Additional file 2. Fig. 6 The abnormal CT findings of the patient. A: The axial image of contrast-enhanced computed tomography scan shows hepatosplenomegaly (asterisk) and intestinal wall thickening (arrow);B: Axial unenhanced computed tomography shows slightly enlarged mesenteric lymph nodes (arrow); C:The axial image of computed tomography scan shows intestinal wall thickening suspicious (arrow); D:Computed tomography with intravenous contrast comfirms the thickened intestinal wall (arrow). [file 12887_2020_2075_MOESM2_ESM.tif]
